# Evaluation of Intestinal Barrier Dysfunction with Serum Iohexol Concentration in Dogs with Acute Hemorrhagic Diarrhea Syndrome

**DOI:** 10.3390/ani14060963

**Published:** 2024-03-20

**Authors:** Andrea Reisinger, Helene Stübing, Patricia E. Ishii, Jan S. Suchodolski, Jonathan A. Lidbury, Kathrin Busch, Stefan Unterer

**Affiliations:** 1 Clinic of Small Animal Internal Medicine, Centre for Clinical Veterinary Medicine, Ludwig-Maximilians-University, 80539 Munich, Germany; 2 Gastrointestinal Laboratory, Department of Small Animal Clinical Sciences, Texas A&M University, College Station, TX 77843, USA; 3 Clinic for Small Animal Internal Medicine, Vetsuisse Faculty, 8057 Zurich, Switzerland

**Keywords:** iohexol, AHDS, intestinal permeability, necrotizing enteritis, SIRS, NetF toxin

## Abstract

**Simple Summary:**

Acute hemorrhagic diarrhea syndrome (AHDS) is characterized by severe gastrointestinal fluid loss and necrotizing enteritis. This indicates an intestinal barrier dysfunction, altered intestinal permeability may indicate disease severity and might represent a risk factor for the development of chronic disorders. Serum iohexol measurement is utilized to assess intestinal permeability in dogs. Our study aimed to correlate intestinal permeability (measured by serum iohexol concentration (SIC)) with clinical severity in dogs with AHDS. We hypothesized increased intestinal permeability in dogs with AHDS, correlating with clinical severity. Fifty-three client-owned dogs, including those with AHDS and healthy controls, were enrolled. Clinical severity was assessed using the AHDS index and criteria for systemic inflammatory response syndrome (SIRS). Simultaneously, dogs were orally administered iohexol, and serum samples were collected for SIC measurement. The findings revealed significantly elevated SIC among dogs with AHDS compared to healthy controls, with a positive correlation between SIC and disease severity indices. These results highlight that dogs with a severe form of AHDS have an especially significant dysfunction of the intestinal barrier. Future studies are necessary to evaluate a potential association between an altered intestinal barrier at the phase of acute enteritis and the development of chronic intestinal disease later in life.

**Abstract:**

Histopathologic examination of intestinal biopsies from dogs with acute hemorrhagic diarrhea syndrome (AHDS) reveals necrotizing enteritis and epithelial integrity loss. Serum iohexol measurement has been utilized to assess intestinal permeability. Our hypothesis is that dogs with AHDS have increased intestinal permeability, which is associated with the severity of clinical signs. In this prospective case–control study, 53 client-owned dogs (28 AHDS, 25 healthy controls) were evaluated. Clinical severity was assessed using the AHDS index and systemic inflammatory response syndrome (SIRS) criteria. Simultaneously, dogs received oral iohexol, and serum iohexol concentrations (SICs) were measured two hours later. Results indicated significantly higher (*p* = 0.002) SIC in AHDS dogs (median: 51 µg/mL; min–max: 9–246) than in healthy controls (30 µg/mL; 11–57). There was a significant positive correlation between AHDS index and SIC (r_S_ = 0.4; *p* = 0.03) and a significant negative between SIC and serum albumin concentrations (Pearson r = −0.55; *p* = 0.01). Dogs with severe AHDS (mean 106 µg/mL; range: 17–246) demonstrated significantly higher (*p* = 0.002) SIC than those with mild to moderate disease (29 µg/mL; 9–54). These findings underscore the association between intestinal permeability and clinical severity in dogs with AHDS assessed by iohexol.

## 1. Introduction

In addition to its role in absorbing water and nutrients, the gastrointestinal tract also functions as a protective barrier against pathogens and harmful substances [1]. The disruption of this barrier results in increased intestinal permeability, leading to increased loss of proteins, electrolytes, vitamins, and fluid into the intestinal lumen and facilitating the translocation of potentially harmful substances and pathogens into the bloodstream [1,2]. As discussed below, there is a suspicion of intestinal barrier dysfunction in dogs with acute hemorrhagic diarrhea syndrome (AHDS).

AHDS is characterized by the sudden onset of hemorrhagic diarrhea [3]. Intestinal biopsies demonstrate the presence of necrotizing enterocolitis, suggesting a likely dysfunction of the mucosal–blood barrier [4]. The loss of albumin through the intestine further supports the hypothesis of intestinal barrier dysfunction in dogs with AHDS [5]. During the acute phase of the disease, intestinal biopsies have revealed the presence of clostridial strains adhering to the surface of necrotic lesions [4]. Recently, a *Clostridium perfringens* type A strain carrying pore-forming toxins (NetE and NetF) was isolated from a dog with AHDS, and the cytotoxic effect of NetF has been confirmed in vitro [6]. Based on these findings and other studies demonstrating a significantly higher occurrence of NetF-toxin-encoding *C. perfringens* in dogs with AHDS compared to healthy dogs or dogs with parvovirus infection [7,8,9], it is suspected that *C. perfringens* and NetF play a major role in the pathogenesis of AHDS. In addition to the toxic effect of NetF, intestinal ischemia, resulting from hypovolemia and hypoperfusion of the intestinal mucosa caused by extensive fluid loss as well as reperfusion damage, may contribute to increased permeability in these dogs [10,11].

Dogs with AHDS often show hypoalbuminemia [5,12]. This not only allows the hypothesis that dogs with AHDS have increased intestinal permeability (IP) but also confirms this with a high degree of probability. However, intestinal permeability (IP) has never really been measured in dogs with AHDS. Various markers are available for assessing IP, but they differ in size, location, degradation mechanism, and analysis method [13]. In addition to endogenous markers for IP measurement (e.g., albumin), there are also numerous exogenous markers. Endogenous markers are often influenced by many factors, such as the degree of dehydration, so these parameters are very variable in individual dogs [12]. Therefore, we focused on exogenous markers for the determination of IP in the study. ^51^Chromium-labeled ethylenediaminetetraacetic acid (^51^Cr-EDTA) is considered the gold standard, but its use is limited due to radioactivity [14,15]. Other IP tests employ sugars like lactulose, sucrose, and rhamnose [16,17]. However, these sugars are metabolized differently and often degraded in the stomach or small intestine in dogs, failing to represent permeability across the entire gastrointestinal tract [18]. In a recent study, iohexol, a non-radioactive iodinated contrast medium, was examined for its potential in assessing intestinal permeability [14,15]. Iohexol has a similar permeability pathway to ^51^Cr-EDTA [14,15] but offers the advantage of not being radioactive. In addition, the serum iohexol concentration can easily be measured using a commercially available enzyme-linked immunosorbent assay (ELISA) rather than complex assays such as liquid chromatography–mass spectrometry [19].

Therefore, we tried to find a measurable way to identify severe barrier dysfunction by evaluating iohexol. That is why the objectives of this study were to evaluate IP by measuring serum iohexol concentrations (SICs) in dogs with AHDS, comparing them with healthy dogs, and establishing correlations between serum iohexol levels, clinical severity (e.g., AHDS index), and SIRS criteria.

## 2. Materials and Methods

### 2.1. Study Design

The prospective case–control study was approved by the Ethics Committee of Veterinary Medicine, Ludwig-Maximilians-University, Munich, Germany (approval number 128-10-06-2018). All dogs included in the study were client-owned and presented to the Clinic for Small Animal Medicine, LMU Munich, Germany between October 2020 and March 2022. The owners were fully informed about the objectives of the study.

### 2.2. Study Population

#### 2.2.1. Dogs with AHDS

Dogs of all breeds, sexes, and ages presenting with acute hemorrhagic diarrhea (<3 days) were eligible for inclusion in the study. Dogs were excluded if they had received medications known to cause mucosal erosions, which could lead to similar symptoms as AHDS (e.g., NSAIDs, corticosteroids, doxycycline), the week before presentation or if they had underlying diseases that could have contributed to hemorrhagic diarrhea. Underlying diseases were suspected if there was evidence of extra-gastrointestinal disorders such as pancreatitis, exocrine pancreatic insufficiency, acute kidney injury, or acute liver failure. Complete blood counts (CBCs) and serum biochemistry were performed on all dogs to assess their overall health status. Dogs with hypoadrenocorticism disease were also excluded. None of the included dogs exhibited hyponatremia, hyperkalemia, or both. Additionally, no dogs displayed an absent stress leukogram. Abdominal ultrasound was conducted on all dogs to screen for focal intestinal disorders (e.g., neoplasia), mechanical obstructions (e.g., foreign body, intussusception), and other visceral diseases. Dogs suspected of having pancreatitis (based on typical ultrasound findings and high-grade abdominal pain) were also excluded. Furthermore, dogs with positive fecal examinations for nematodes (flotation) or protozoan parasites (flotation and IDEXX SNAP Giardia test kit, IDEXX GmbH, Kornwestheim, Germany) were not included. Standardized treatment was administered to all dogs, consisting of fluid therapy (crystalloids, with fluid volume adjusted according to dehydration grade, maintenance requirements, and ongoing losses), analgesics (buprenorphine 0.01 mg/kg i.v. every 6–8 h or metamizole 50 mg/kg i.v. every 8 h), and antiemetics (maropitant 1 mg/kg i.v. every 24 h, with metoclopramide 60 μg/kg/h i.v. as needed). Antibiotic treatment was given to seven out of the twenty-eight dogs. One dog received marbofloxacin at a dosage of 4 mg/kg i.v. for 1 day, followed by 2 mg/kg i.v. every 24 h, while five dogs were treated with amoxicillin-clavulanic acid at a dosage of 20 mg/kg i.v. every 8 h. In all of these cases, antibiotic treatment was initiated due to suspected sepsis, as determined by the attending veterinarian. An additional dog received amoxicillin-clavulanic acid at a dosage of 12.5 mg/kg i.v. every 8 h due to a urinary infection and a nephrolith. Antibiotic treatment in all seven dogs began after sampling for iohexol measurement. After the intervention, a subset of dogs (n = 14) was included in another study evaluating microbiota modulation and therefore was not included in the statistical correlation analysis between SIC and length of hospitalization time. Dogs were eligible for discharge from the hospital when they exhibited a good general condition and when their diarrhea was no longer bloody, with a frequency of ≤3 times per day, indicating that fluid losses no longer required intravenous fluid administration.

#### 2.2.2. Healthy Control Group

The control group comprised 25 client-owned dogs that were clinically healthy. Fecal testing (Giardia antigen test; fecal flotation) was conducted on all dogs to exclude Giardia and other endoparasites. Furthermore, comprehensive assessments including CBC, serum biochemistry, vitamin B12, folic acid, basal cortisol, canine trypsin-like immunoreactivity (cTLi), and canine pancreatic lipase immunoreactivity (cPLi) were performed on all dogs. Dogs were included in the study if all the above tests were negative or within the reference interval.

### 2.3. Iohexol Measurement

Following rehydration, a mixture of 2 mL/kg body weight of iohexol (OmnipaqueTM 350 mg/mL, GE Healthcare, Munich, Germany) and food (gastrointestinal liquid diet, Royal Canin, Cologne, Germany) was administered orally to the dogs using a syringe. Serum samples were collected 2 h after iohexol administration [14]. It is important to note that if any of the dogs had vomited at the time of the SIC measurement, they would have been excluded from the study. These samples were then frozen and stored at −80 °C until the measurement of SIC, which was performed using a previously validated ELISA (Functional immunoassay technology (FIT)-GFRTM Iohexol kit, BioPAL, Worcester, MA, USA) [19]. The same procedure was carried out for the healthy dogs.

### 2.4. Evaluation of Disease Severity and Clinical Outcome

Disease severity was assessed using the AHDS index (Table 1) [12,20] on the day of presentation and concurrently with the iohexol measurement. Based on the AHDS index, dogs were categorized into two groups: dogs with mild–moderate (AHDS index < 9) and dogs with severe disease activity (AHDS index ≥ 9). This allowed for the correlation between IP and clinical severity. Fecal consistency was assessed using the Purina Fecal Scoring System for Dogs (PFS), which can be accessed at https://www.purina.de/artikel/hunde/gesundheit/verdauung/hundekot (accessed on 8 August 2022). Signs of systemic disease were evaluated based on criteria for systemic inflammatory response syndrome (SIRS) (Table 2). These criteria were adapted from previous veterinary publications [21,22,23]. Hypoglycemia and the presence of an inflammatory leukogram were additional factors considered for SIRS categorization. CBCs were not performed in eight out of twenty-eight dogs, and glucose measurements were not performed in seven out of twenty-eight dogs at the time of SIRS criteria determination. The severity of the disease according to the SIRS criteria was determined at the time of iohexol measurement, when the dogs had already been rehydrated to minimize any potential interference of hypovolemia with clinical signs of SIRS [22]. In addition, the serum iohexol concentration was measured during the determination of the SICs. A serum albumin concentration of <30 g/L was classified as hypoalbuminemia.

### 2.5. Statistical Analysis

Statistical analyses were performed using Microsoft Office Excel version 16.63.1 and GraphPad Prism version 9.4.1 (San Diego, CA, USA). Normal distribution was tested using the Shapiro–Wilk normality test. The Mann–Whitney U-test and unpaired *t*-test were employed to compare SIC between healthy dogs and dogs with AHDS, as well as to assess the relationship between SIC and clinical severity indicators (AHDS index, SIRS criteria, hypoalbuminemia). Therefore, the SICs are expressed below as mean or median according to the normal distribution. Spearman’s rank correlation (r_s_) and Pearson’s correlation coefficient (r_P_) were used to examine the correlation between SIC and albumin concentration, hospitalization time, and AHDS index. A *p*-value of <0.05 was considered statistically significant.

## 3. Results

### 3.1. Signalment

A total of 28 dogs with AHDS met the inclusion criteria. The mean age of dogs with AHDS was 7.0 years (range: 1.2–14.1 years). The most represented breeds were mixed-breed dogs (10), Cavalier King Charles Spaniel (2), Shih Tzu (2), Cocker Spaniel (2), and Dachshund (2). Fifteen dogs were female (six spayed), and thirteen dogs were male (six neutered). The mean body weight was 11.2 kg (range: 1.8–46). In the control group, there were 25 healthy dogs with a mean age of 4.7 years (range: 1–10 years). The most common breeds were mixed-breed dogs (7). Seventeen dogs were male, and eight dogs were female. The mean body weight was 16.4 kg (range: 2.6–43.3). There was a significant difference between the two groups in age (*p* = 0.02). The weight did not differ significantly between the two groups (*p* = 0.96). With regard to age, there was no significant difference between dogs with AHDS that had SIC > 30 μg/mL and dogs with AHDS that had SIC < 30 μg/mL (*p* = 0.26). A SIC of 30 μg/mL was chosen as the cut-off because this was the median value of SIC in the healthy control group (see below).

### 3.2. Comparison of SIC between the Healthy Control Group and Dogs with AHDS

**Figure 1:**
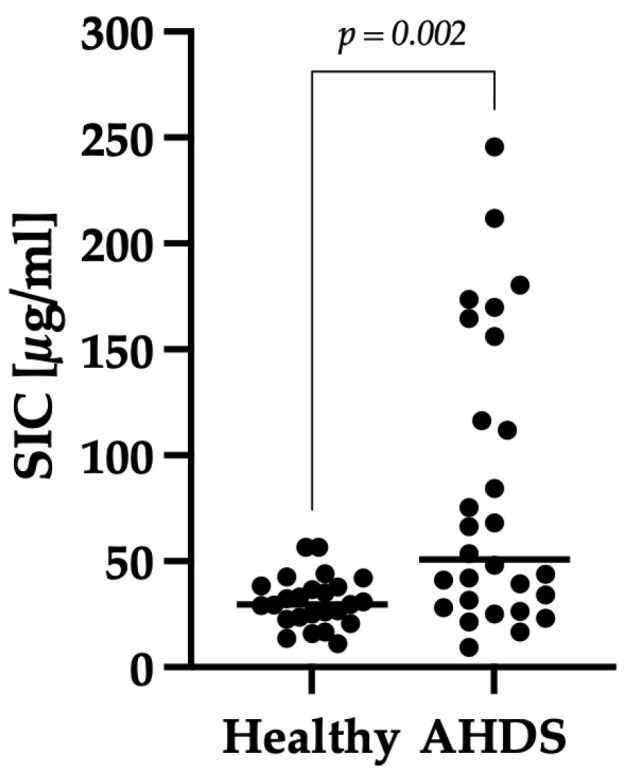
Comparison of SIC of healthy dogs and dogs with AHDS. The y-axis shows the level of SIC in μg/mL. The horizontal lines represent the median SIC. Dogs with AHDS showed significantly higher SIC compared to healthy dogs (*p* = 0.002).

Dogs with AHDS had significantly higher SIC than those in the healthy control group (*p* = 0.002). The dogs with AHDS had a median SIC value of 51 μg/mL (min–max: 9–246), whereas the healthy control group had a median SIC value of 30 μg/mL (min–max: 11–57) (Figure 1).

### 3.3. Correlation between Clinical Severity and Serum Iohexol Concentration

**Figure 2:**
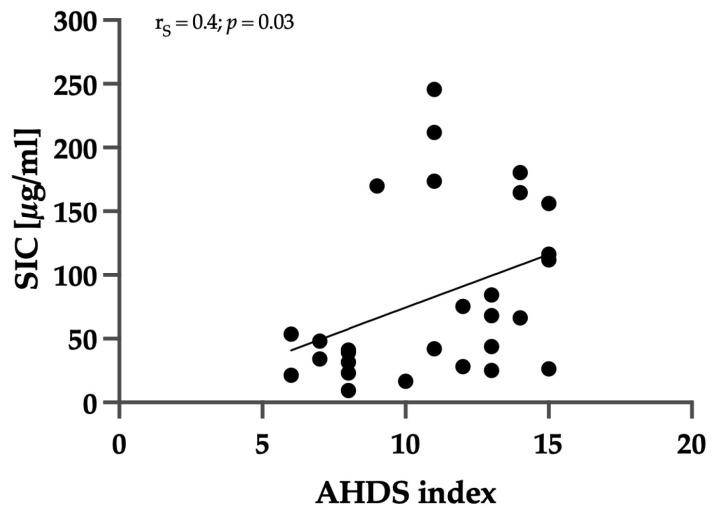
Correlation between AHDS index and SIC. The AHDS index includes the parameters activity, appetite, vomiting, fecal consistency, fecal frequency, and dehydration grade. Each parameter has a score of 0–3, and the sum of scores gave a total cumulative score. The AHDS index was determined similarly to the SIC. There was a significant positive correlation between the AHDS index and SIC (r_S_ = 0.4; *p* = 0.03).

Nine of twenty-eight dogs were classified into the group with mild–moderate (AHDS index < 9) and nineteen of twenty-eight with severe disease activity (AHDS index ≥ 9). Seven out of the twenty-eight dogs exhibited significant hemoconcentration, with a hematocrit level greater than 60% at the time of presentation. There was no significant difference in SIC between dogs with a hematocrit ≤60% and >60% (*p* = 0.5). A significant positive correlation was observed between the AHDS index and SIC (r_S_ = 0.4; *p* = 0.03), as shown in Figure 2.

**Figure 3:**
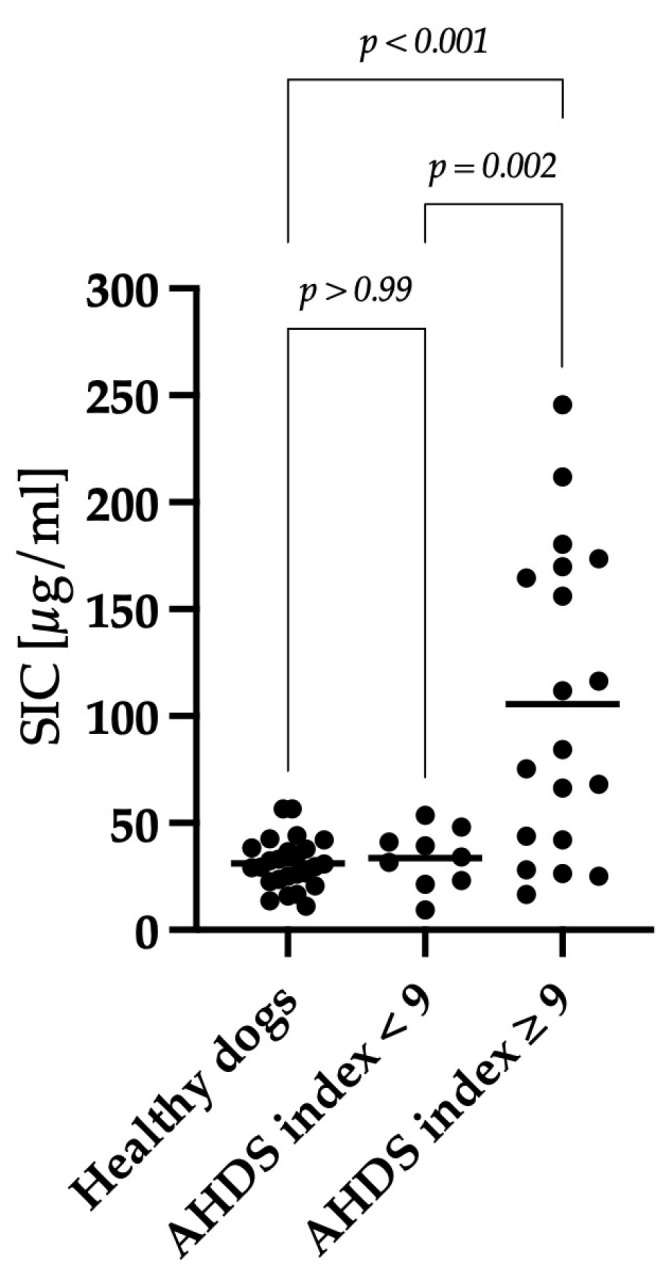
Comparison of SIC between healthy dogs, dogs with a mild–moderate clinical course, and dogs with a severe clinical course. Dogs with an AHDS index ≥ 9 (n = 19) showed significantly higher SIC compared to dogs clinically classified as mild–moderate (AHDS index < 9; n = 9) (*p* = 0.002) and healthy dogs (n = 25) (*p* < 0.001). There was no difference in SIC between healthy dogs and the dogs with a mild–moderate disease activity (*p* > 0.99). The horizontal lines show the mean SIC in each group.

Healthy dogs exhibited a mean SIC of 31 μg/mL (range: 11–57), dogs with a mild–moderate disease activity of 29 μg/mL (range: 9–54) and dogs with a severe disease activity of 106 μg/mL (range: 17–246). There was no significant difference in SIC between healthy dogs and dogs with a mild–moderate disease activity (*p* > 0.99). However, dogs classified with severe clinical activity displayed significantly higher SIC levels compared to healthy dogs (*p* < 0.001) and dogs classified with low to moderate clinical activity (*p* = 0.002), as depicted in Figure 3.

It is important to note that while all 28 dogs survived, the correlation between SIC and hospitalization time only included 14 out of 28 dogs, as the remaining dogs were part of a separate study focused on evaluating microbiota modulation. The median hospitalization time for dogs with AHDS was 3 days. A significant positive correlation was found between hospitalization time and SIC (r_S_ = 0.69; *p* = 0.007). There was no significant correlation (r_P_ = 0.29 *p* = 0.3) between hospitalization time and AHDS index.

**Figure 4:**
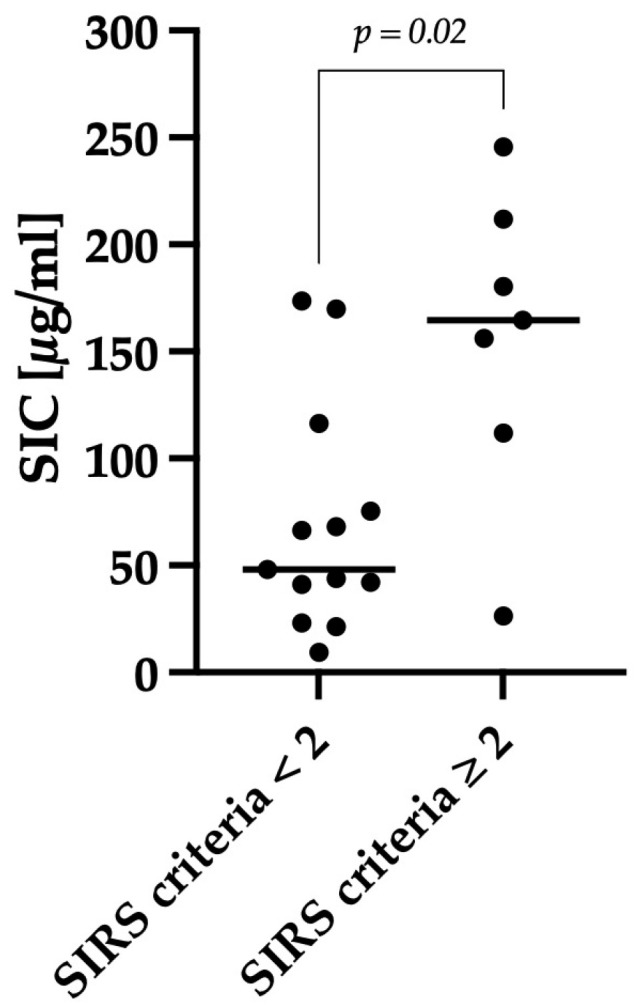
Comparison of SIC between dogs classified as positive for the occurrence of SIRS and dogs classified as negative. SIRS criteria were assessed after rehydration at the time of SIC measurement. The criteria included clinical criteria (hypo- or hyperthermia, tachycardia, and tachypnea) and laboratory changes (leukopenia or leukocytosis, left shift, hypoglycemia). If a dog had ≥ 2 criteria, it was classified as positive for the occurrence of SIRS. These dogs (n = 7) showed significantly (*p* = 0.02) higher SIC compared with dogs classified as negative for the occurrence of SIRS (n = 13). The horizontal lines show the median SIC of the different groups.

Dogs that met two or more SIRS criteria after rehydration and pain management were classified as having a systemic inflammatory response. A significant difference in SIC (*p* = 0.02) was observed between dogs with two or more SIRS criteria (n = 7; median: 165; min–max: 26–246) and dogs with less than two SIRS criteria (n = 13; median: 48; min–max: 9–174), as shown in Figure 4.

**Figure 5:**
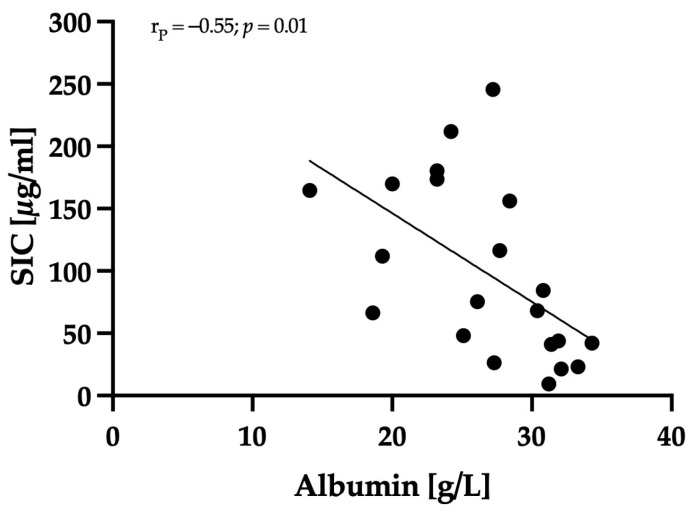
Correlation of serum albumin concentration and SIC. The serum albumin concentration was determined in 21/28 dogs after rehydration. The serum albumin concentration correlated significantly negatively (r_P_ = −0.55; *p* = 0.01) with SIC.

Serum albumin concentration was determined after rehydration in 21 out of 28 dogs, and it exhibited a significant negative correlation with SIC (r_P_ = −0.55; *p* = 0.01); see Figure 5.

**Figure 6:**
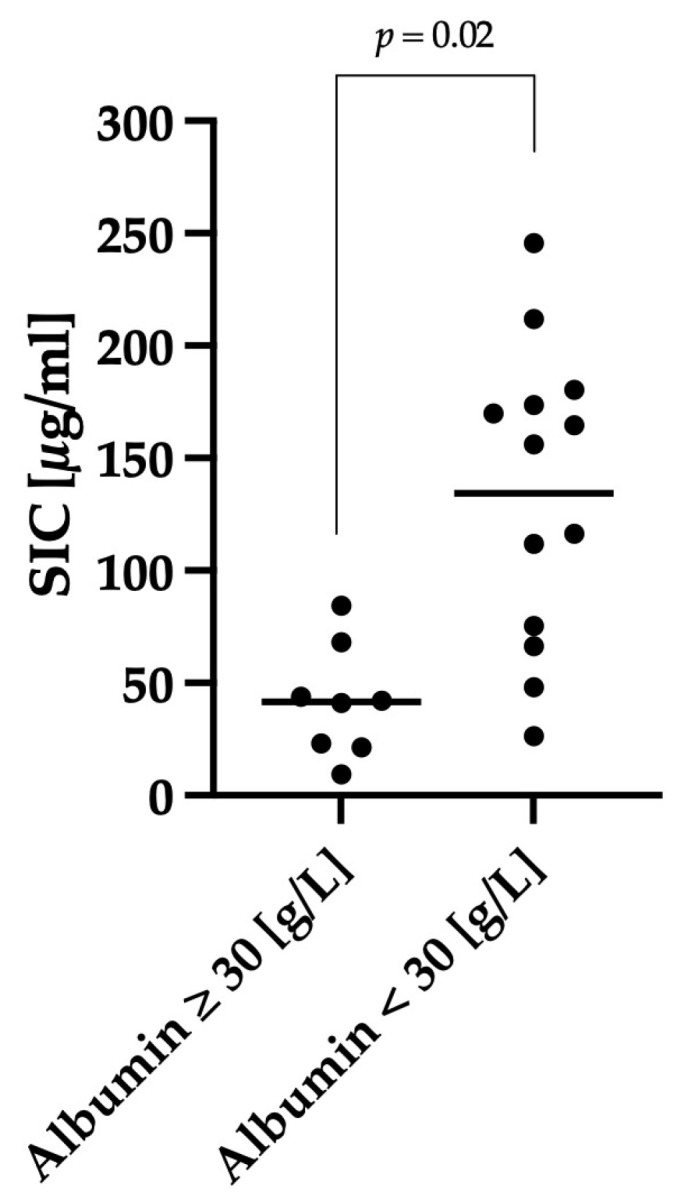
Comparison of SIC between dogs that were hypoalbuminemic and dogs that were not. We defined a serum albumin concentration < 30 g/L as hypoalbuminemia. Dogs with hypoalbuminemia (n = 13) showed significantly (*p* = 0.02) higher SIC compared with dogs with a normal albumin concentration ≥ 30 g/L (n = 8). The horizontal lines show the mean SIC of the different groups.

Hypoalbuminemia was defined as a serum albumin concentration < 30 g/L. Dogs with AHDS and hypoalbuminemia (n = 13; mean: 134; range: 26–246) had significantly higher SIC levels (*p* = 0.02) compared to dogs with albumin concentrations within the reference range (n = 8; mean: 42; range: 9–84) (Figure 6).

### 3.4. Antibiotic Group

**Figure 7:**
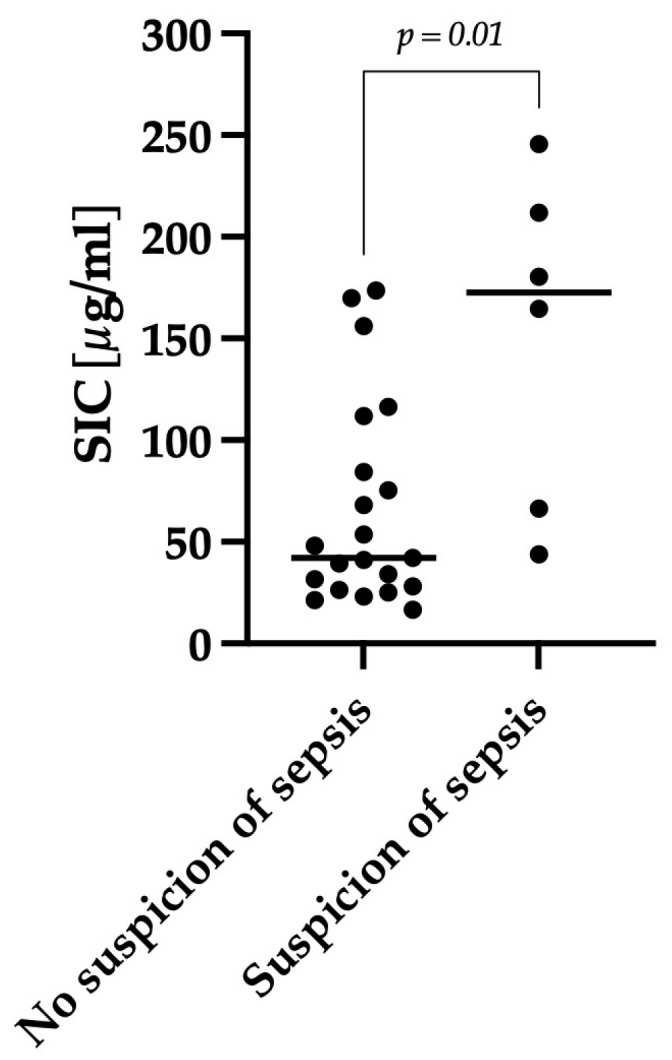
Comparison of SIC between dogs with AHDS with and without antibiotic treatment due to the suspicion of sepsis. Dogs in which sepsis was suspected (n = 6) showed significantly higher SIC compared to other dogs with AHDS (n = 21) (*p* = 0.01). The horizontal lines show the median SIC of the two different groups.

Out of the twenty-eight dogs, seven received antibiotic treatment. Among these, six dogs were treated because the attending veterinarian classified them as septic. One dog received antibiotics due to an incidental finding of a urinary infection and a nephrolith, and this particular dog was excluded from the subsequent analysis as the antibiotic use was unrelated to the severity of AHDS. In all dogs, antibiotic treatment was initiated after sampling for iohexol measurement. Dogs in which a sepsis was suspected, and which received antibiotics, exhibited significantly higher SIC levels (*p* = 0.01) compared to the other dogs with AHDS, as shown in Figure 7. Dogs suspected as septic (n = 6) had a median SIC value of 173 μg/mL (min–max: 44–246), while the other dogs with AHDS (n = 21) had a median SIC value of 42 μg/mL (min–max: 17–174). All six dogs had an AHDS index ≥ 9, indicating severe disease. Moreover, four out of six dogs were classified as positive for the presence of SIRS, and five out of six dogs were hypoalbuminemic. Follow-up was possible for 14 out of 28 dogs, revealing a significant difference (*p* = 0.01) in hospitalization time between dogs that were treated with antibiotics due to suspicion of sepsis (n = 3) and other dogs with AHDS (n = 11).

## 4. Discussion

This study assessed IP in dogs with AHDS by measuring SICs. Our findings revealed an elevated IP when compared to a healthy control group. Additionally, we observed a correlation between the severity of clinical signs in dogs with AHDS and the extent of IP.

IP refers to the ability of substances to traverse the intestinal wall and enter the bloodstream. Various factors can disrupt the integrity of the intestinal barrier, resulting in increased IP [1,2,24,25]. These factors encompass infiltration of neoplastic or inflammatory cells into the mucosa and exposure to toxins [1,2,24,25]. In humans, infections caused by toxin-producing bacteria (e.g., Vibrio cholerae’s cholera toxin, *Escherichia coli* O157:H7′s Shiga toxin) can inflict substantial damage to the intestinal epithelium [26,27]. Given the occurrence of necrotizing enteritis observed in histopathologic studies, we postulated that dogs with AHDS might exhibit increased IP, likely due to a breakdown of the mucosa–blood barrier [4]. This breakdown is thought to be attributable to the presence of toxins, particularly NetF, produced by *C. perfringens*, leading to epithelial destruction [4,6,7,9,28]. Additionally, severe dehydration resulting from prolonged diarrhea could contribute to heightened permeability [10,12,22]. The resultant reduced perfusion of splanchnic organs may further exacerbate increased IP. Assessing IP in dogs with AHDS could be beneficial for gauging disease severity. Specifically, it may provide insights into nutrient malabsorption, the absorption of orally administered drugs, and the identification of an elevated risk of bacterial and antigen translocation, potentially culminating in sepsis and immune system sensitization.

Multiple techniques are available for measuring IP. In our study, we employed iohexol to determine IP. Iohexol follows a similar pathway to the gold standard marker, ^51^Cr-EDTA, but offers the advantage of being non-radioactive, making it suitable for routine clinical use [14,17,29,30]. Its efficacy has been demonstrated in previous research, further supporting its utilization [14,15]. Unlike sugars such as rhamnose, sucrose, and lactose, which are commonly employed permeability markers, iohexol remains unaffected by bacterial degradation or metabolism, enabling permeability assessment across the entire gastrointestinal tract [15,17]. Additionally, iohexol exhibits low osmolarity, binds minimally to plasma proteins, and demonstrates low toxicity and enhanced tolerability [31,32,33,34]. Its hydrophilic nature restricts easy traversal across lipophilic cell membranes [31]. Studies have consistently affirmed iohexol as a reliable and accurate marker for evaluating IP [14,15,29,34,35]. In human medicine, elevated SIC has been observed in patients with Crohn’s disease compared to healthy individuals, with a notable positive correlation between clinical severity and SIC [36]. In dogs, iohexol has been utilized to determine glomerular filtration rate in previous investigations [37,38,39]. Two specific studies have assessed IP using iohexol: one conducted by Frias et al., which demonstrated a strong linear association between iohexol and ^51^Cr-EDTA in healthy beagle dogs [15], and another conducted by Klenner et al., which examined different dosages and the timing of serum iohexol determination in healthy dogs [14]. Following the findings of Klenner et al., we administered an oral dose of 2 mL/kg of iohexol and measured SIC after a 2 h interval, as this yielded the highest SIC in healthy dogs [14].

In our study, dogs with AHDS exhibited significantly higher SIC than dogs in the healthy control group. However, it was surprising that only a subset of AHDS patients displayed increased SIC compared to the healthy dogs. One possible explanation could be variations in the severity of mucosal barrier destruction among patients, leading to varying degrees of IP. The clinical progression of AHDS is highly dynamic, with most dogs experiencing clinical improvement within a few days, while a smaller proportion may develop complications such as significant hypoalbuminemia and signs of systemic inflammation. Additionally, the severity and extent of histologic lesions can vary significantly among dogs with AHDS [40]. These factors, along with variations in disease severity and different stages of presentation, might account for the differences observed in SIC or for the fact that SIC may not have optimal sensitivity for IP.

We evaluated clinical severity using the AHDS index and SIRS criteria and found a significant correlation between SIC and the AHDS index. This implies that dogs with more pronounced clinical signs exhibited a greater increase in IP. While it can be presumed that all dogs with hemorrhagic diarrhea have some level of mucosal damage, elevated SIC may specifically indicate dogs with severe mucosal destruction. Considering iohexol’s larger molecular size (821 Dalton) compared to ^51^Cr-EDTA (359 Dalton), iohexol may still be too large to detect mild forms of IP changes. Further studies employing ^51^Cr-EDTA, a more sensitive marker of IP, could be beneficial in identifying even minor functional changes in this group of dogs.

Furthermore, dogs with hypoalbuminemia (<30 g/L) exhibited significantly higher SIC than dogs with normal serum albumin levels. However, it is worth noting that there was one dog with hypoalbuminemia that demonstrated a SIC of 26 μg/mL, which is lower than the median SIC of healthy dogs. This finding was unexpected since albumin, with a molecular weight of 66,470 Daltons, is considerably larger than iohexol (821 Daltons), which, due to its smaller size, should more easily pass the intestinal barrier. One possible explanation is that SIC measurements offer only a transient snapshot, and it is plausible that serum iohexol concentrations in these dogs could have been higher at a different time point of measurement. We tried to standardize the SIC measurement, but we cannot rule out the possibility that some individual dogs had a delayed gastric emptying due to disease-associated hypomotility and thus less iohexol arrived in the small intestine, the region where it is absorbed in case of barrier dysfunction.

There are inherent risks of sepsis development in dogs with AHDS [10]. Alongside the presence of a damaged mucosal surface, numerous bacteria can be found adhering to the necrotic surface [4], potentially representing an additional risk factor for bacterial translocation. Furthermore, dogs with AHDS commonly experience severe dehydration, which further contributes to the likelihood of sepsis development [10]. In our study, SIRS assessment was conducted after rehydrating dogs with AHDS to prevent misclassification due to hypovolemia. Dogs that met two or more SIRS criteria demonstrated significantly higher SIC compared to dogs without evidence of SIRS. This result indicates that the severity of clinical signs is associated with the degree of IP. Despite the general risk of bacterial translocation, most dogs with AHDS exhibit rapid improvement with symptomatic treatment alone. The administration of antibiotics did not result in a significantly faster resolution of clinical signs in a group of dogs with aseptic AHDS [22]. In our study, six dogs received antibiotic treatment due to suspected sepsis, and these dogs displayed significantly higher SIC than the other dogs. Additionally, these dogs were classified as clinically severe (AHDS index ≥ 9). Furthermore, three of these dogs exhibited the highest SIC (180 μg/mL, 212 μg/mL, 246 μg/mL), indicating the highest degree of IP. These findings might support the hypothesis that severe intestinal barrier dysfunction could lead to an increased translocation of intestinal bacteria, leading to SIRS symptoms. To confirm this with greater certainty, blood cultures or the measurement of lipopolysaccharides would have been necessary, as it is conceivable that these dogs did not exhibit sepsis, but only severe SIRS, and therefore, antibiotic therapy would not have been indispensable in these patients. Further studies would be necessary to evaluate whether iohexol can serve as a useful marker in identifying dogs with severe barrier dysfunction that might benefit from antibiotic treatment at presentation prior to fluid therapy.

In a retrospective longitudinal study, dogs with a prior episode of AHDS exhibited a higher prevalence of chronic gastrointestinal signs later in life than control dogs [25]. Many of these dogs responded well to dietary trials, suggesting that allergens crossing the epithelial barrier during the acute disease phase may contribute to the development of food allergies [25]. One possible explanation is that increased IP leads to a heightened transfer of dietary components into the bloodstream. This overwhelms the immune system, resulting in a loss of oral tolerance and subsequent sensitization to food components [25,41]. Further research is required to determine whether this particular subgroup of dogs, characterized by signs of intestinal barrier dysfunction indicated by elevated SIC, faces an elevated risk of developing chronic gastrointestinal disorders. Additionally, investigating whether iohexol can serve as a long-term prognostic marker in dogs with severe hemorrhagic enteritis would be valuable. Alternatively, it is plausible that intestinal damage persists in these patients, leading to changes consistent with chronic inflammatory enteropathy.

One limitation of the study is the variation in treatment among patients. However, it is important to note that none of the dogs received any drugs (such as NSAIDs, corticosteroids, or doxycycline) known to potentially harm the intestinal barrier. Another limitation of the study is that the healthy dogs differed significantly in age from the dogs with AHDS. This limitation was not considered critical by the authors as all dogs were adults, and there was no statistically significant difference in age between dogs with AHDS that had SICs > 30 μg/mL (which was the median value of the healthy control group) and those that had SICs < 30 μg/mL. It can therefore be concluded that age has no significant influence on intestinal permeability.

Our findings support the hypothesis that some dogs with AHDS exhibit heightened IP, as measured by iohexol. However, it is important to note that not all dogs demonstrated increased IP using this marker, which could be attributed to variations in the extent of intestinal mucosal damage. Moreover, we observed a correlation between the degree of IP and clinical severity, with dogs classified as SIRS patients displaying the highest SIC. Follow-up studies are necessary to monitor these patients and determine whether dogs with elevated SIC are at risk of developing chronic gastrointestinal disorders. This investigation will help assess the potential of iohexol as a long-term prognostic marker in dogs with AHDS.

## 5. Conclusions

In conclusion, our study has shown that there is a correlation between increased intestinal permeability, as measured by serum iohexol concentration, and clinical severity in dogs with AHDS. It has also been shown that in general, dogs with AHDS have increased intestinal permeability assessed by SIC. However, further research is needed to evaluate the potential long-term effects of altered intestinal barrier function on the development of chronic intestinal disease in affected dogs.

## Figures and Tables

**Table 1:** Criteria to evaluate the Canine Acute Hemorrhagic Diarrhea Severity (AHDS) Index. Each parameter has a score of 0–3, and the sum of scores gave a total cumulative score. AHDS total index 0 to 3: insignificant disease; 4 to 5: mild disease; 6 to 8: moderate disease; ≥9: severe disease [12,20]. The Purina Fecal Scoring System for Dogs (PFS) was used for fecal consistency.

	0	1	2	3
Activity	Normal	Mildly decreased	Moderately decreased	Severely decreased
Appetite	Normal	Mildly decreased	Moderately decreased	Severely decreased
Fecal consistency	PFS 1–2	PFS 3–4	PFS 5–6	PFS 7
Fecal frequency (times/day)	1	2–3	4–5	>5
Vomiting (times/day)	0	1	2–3	>3
Dehydration (%)	0	<5	5–10	>10

**Table 2:** Inflammatory response syndrome (SIRS) criteria for assessing disease severity in dogs, adjusted from Chu et al. [21] Hauptman et al. [23], and Dupont et al. [22].

Clinical SIRS Criteria	
Hypo- or hyperthermia	<37.5 °C or >39.3 °C <99.5 °F or >102.74 °F
Tachycardia	>140 beats/min
Tachypnea	>40 breaths/min
Laboratory SIRS criteria	
Leukopenia or leukocytosis	<6 G/L or >25 G/L
Band neutrophils	>3%
Hypoglycemia	<70.2 mg/dL or <3.9 mmol/L

## Data Availability

Data available on request due to restrictions (e.g., privacy, legal or ethical reasons).

## References

[1] Konig J., Wells J., Cani P.D., García-Ródenas C.L., MacDonald T., Mercenier A., Whyte J., Troost F., Brummer R.J. (2016). Human Intestinal Barrier Function in Health and Disease. Clin. Transl. Gastroenterol..

[2] Deitch E.A. (1993). Nutrition and the gut mucosal barrier. Curr. Opin. Gen. Surg..

[3] Unterer S.H.K. (2009). Akuter blutiger Durchfall beim Hund—Ursachen und diagnostische Aufarbeitung. Tierarztl. Prax. Ausg. K. Kleintiere Heimtiere.

[4] Unterer S., Busch K., Leipig M., Hermanns W., Wolf G., Straubinger R.K., Mueller R.S., Hartmann K. (2014). Endoscopically visualized lesions, histologic findings, and bacterial invasion in the gastrointestinal mucosa of dogs with acute hemorrhagic diarrhea syndrome. J. Vet. Intern. Med..

[5] Will K., Nolte I., Zentek J. (2005). Early enteral nutrition in young dogs suffering from haemorrhagic gastroenteritis. J. Vet. Med. A Physiol. Pathol. Clin. Med..

[6] Mehdizadeh Gohari I., Parreira V.R., Nowell V.J., Nicholson V.M., Oliphant K., Prescott J.F. (2015). A novel pore-forming toxin in type A *Clostridium perfringens* is associated with both fatal canine hemorrhagic gastroenteritis and fatal foal necrotizing enterocolitis. PLoS ONE.

[7] Sindern N., Suchodolski J.S., Leutenegger C.M., Mehdizadeh Gohari I., Prescott J.F., Proksch A.L., Mueller R.S., Busch K., Unterer S. (2019). Prevalence of *Clostridium perfringens* netE and netF toxin genes in the feces of dogs with acute hemorrhagic diarrhea syndrome. J. Vet. Intern. Med..

[8] Zhang Z., Mocanu V., Cai C., Dang J., Slater L., Deehan E.C., Walter J., Madsen K.L. (2019). Impact of Fecal Microbiota Transplantation on Obesity and Metabolic Syndrome-A Systematic Review. Nutrients.

[9] Ziese A.L., Suchodolski J.S., Hartmann K., Busch K., Anderson A., Sarwar F., Sindern N., Unterer S. (2018). Effect of probiotic treatment on the clinical course, intestinal microbiome, and toxigenic *Clostridium perfringens* in dogs with acute hemorrhagic diarrhea. PLoS ONE.

[10] Unterer S., Lechner E., Mueller R.S., Wolf G., Straubinger R.K., Schulz B.S., Hartmann K. (2015). Prospective study of bacteraemia in acute haemorrhagic diarrhoea syndrome in dogs. Vet. Rec..

[11] Gonzalez L.M., Moeser A.J., Blikslager A.T. (2015). Animal models of ischemia-reperfusion-induced intestinal injury: Progress and promise for translational research. Am. J. Physiol. Gastrointest. Liver Physiol..

[12] Mortier F., Strohmeyer K., Hartmann K., Unterer S. (2015). Acute haemorrhagic diarrhoea syndrome in dogs: 108 cases. Vet. Rec..

[13] Travis S., Menzies I. (1992). Intestinal permeability: Functional assessment and significance. Clin. Sci..

[14] Klenner S., Coenen M., Failing K., Hewicker-Trautwein M., Ternes W., Verspohl J., Spillmann T. (2009). Estimation of intestinal permeability in healthy dogs using the contrast medium iohexol. Vet. Clin. Pathol..

[15] Frias R., Strube K., Ternes W., Collado M.C., Spillmann T., Sankari S., Westermarck E. (2012). Comparison of 51chromium-labeled ethylenediamine tetra-acetic acid and iohexol as blood markers for intestinal permeability testing in Beagle dogs. Vet. J..

[16] Davis M.S., Willard M.D., Williamson K.K., Steiner J.M., Williams D.A. (2005). Sustained strenuous exercise increases intestinal permeability in racing Alaskan sled dogs. J. Vet. Intern. Med..

[17] Steiner J.M., Williams D.A., Moeller E.M. (2002). Kinetics of urinary recovery of five sugars after orogastric administration in healthy dogs. Am. J. Vet. Res..

[18] Meddings J.B., Sutherland L.R., Byles N.I., Wallace J.L. (1993). Sucrose: A novel permeability marker for gastroduodenal disease. Gastroenterology.

[19] Ortín-Piqueras V.S.T., Pöytäkangas M., Vaccaro D.E., Sankari S., Frías R. (2018). Determination of iohexol in canine plasma—Strong correlation between enzyme-linked immunosorbent assay, high- performance liquid chromatography, and neutron activation analysis. Scand. J. Lab. Anim. Sci..

[20] Busch K., Suchodolski J.S., Kuhner K.A., Minamoto Y., Steiner J.M., Mueller R.S., Hartmann K., Unterer S. (2015). *Clostridium perfringens* enterotoxin and Clostridium difficile toxin A/B do not play a role in acute haemorrhagic diarrhoea syndrome in dogs. Vet. Rec..

[21] Chu V., Goggs R., Bichoupan A., Radhakrishnan S., Menard J. (2021). Hypophosphatemia in Dogs With Presumptive Sepsis: A Retrospective Study (2008–2018). Front. Vet. Sci..

[22] Dupont N., Jessen L.R., Moberg F., Zyskind N., Lorentzen C., Bjørnvad C.R. (2021). A retrospective study of 237 dogs hospitalized with suspected acute hemorrhagic diarrhea syndrome: Disease severity, treatment, and outcome. J. Vet. Intern. Med..

[23] Hauptman J.G., Walshaw R., Olivier N.B. (1997). Evaluation of the sensitivity and specificity of diagnostic criteria for sepsis in dogs. Vet. Surg..

[24] Sun Z., Wang X., Andersson R. (1998). Role of intestinal permeability in monitoring mucosal barrier function. History, methodology, and significance of pathophysiology. Dig. Surg..

[25] Skotnitzki E., Suchodolski J.S., Busch K., Werner M., Zablotski Y., Ballhausen B.D., Neuerer F., Unterer S. (2022). Frequency of signs of chronic gastrointestinal disease in dogs after an episode of acute hemorrhagic diarrhea. J. Vet. Intern. Med..

[26] Melton-Celsa A., Mohawk K., Teel L., O’Brien A. (2012). Pathogenesis of Shiga-toxin producing escherichia coli. Curr. Top. Microbiol. Immunol..

[27] Crisan C.V., Hammer B.K. (2020). The Vibrio cholerae type VI secretion system: Toxins, regulators and consequences. Environ. Microbiol..

[28] Mehdizadeh Gohari I., Unterer S., Whitehead A.E., Prescott J.F. (2020). NetF-producing *Clostridium perfringens* and its associated diseases in dogs and foals. J. Vet. Diagn. Investig..

[29] Rummell L.M., Steele M.A., Templeman J.R., Yohe T.T., Akhtar N., Lambie J.G., Singh P., Asquith T., Verbrugghe A., Pearson W. (2022). A proof of principle study investigating the effects of supplemental concentrated brewer’s yeast on markers of gut permeability, inflammation, and fecal metabolites in healthy non-challenged adult sled dogs. J. Anim. Sci..

[30] Forsgard R.A., Korpela R., Holma R., Lindén J., Frias R., Spillmann T., Österlund P. (2016). Intestinal permeability to iohexol as an in vivo marker of chemotherapy-induced gastrointestinal toxicity in Sprague-Dawley rats. Cancer Chemother. Pharmacol..

[31] Mutzel W., Speck U. (1980). Pharmacokinetics and biotransformation of iohexol in the rat and the dog. Acta Radiol. Suppl..

[32] Lasser E.C., Farr R.S., Fujimagari T., Tripp W.N. (1962). The significance of protein binding of contrast media in roentgen diagnosis. Am. J. Roentgenol. Radium Ther. Nucl. Med..

[33] Bettmann M.A., Morris T.W. (1986). Recent advances in contrast agents. Radiol. Clin. N. Am..

[34] Strube K. (2007). Vergleichende Iohexol- und Iodbestimmung in Caninen und Equinen Serum- und Rattenurinproben Nach Oraler Verabreichung von Iohexol—Ein Beitrag zum Möglichen Einsatz von Iohexol als Marker Für Die Intestinale Permeabilität. Ph.D. Thesis.

[35] Ortin-Piqueras V., Freitag T.L., Andersson L.C., Lehtonen S.H., Meri S.K., Spillmann T., Frias R. (2021). Urinary Excretion of Iohexol as a Permeability Marker in a Mouse Model of Intestinal Inflammation: Time Course, Performance and Welfare Considerations. Animals.

[36] Gerova V.A., Stoynov S.G., Katsarov D.S., Svinarov D.A. (2011). Increased intestinal permeability in inflammatory bowel diseases assessed by iohexol test. World J. Gastroenterol..

[37] Finco D.R., Braselton W.E., Cooper T.A. (2001). Relationship between plasma iohexol clearance and urinary exogenous creatinine clearance in dogs. J. Vet. Intern. Med..

[38] Laroute V., Lefebvre H.P., Costes G., Toutain P.L. (1999). Measurement of glomerular filtration rate and effective renal plasma flow in the conscious beagle dog by single intravenous bolus of iohexol and p-aminohippuric acid. J. Pharmacol. Toxicol. Methods.

[39] Baklouti S., Concordet D., Borromeo V., Pocar P., Scarpa P., Cagnardi P. (2021). Population Pharmacokinetic Model of Iohexol in Dogs to Estimate Glomerular Filtration Rate and Optimize Sampling Time. Front. Pharmacol..

[40] Leipig-Rudolph M., Busch K., Prescott J.F., Mehdizadeh Gohari I., Leutenegger C.M., Hermanns W., Wolf G., Hartmann K., Verspohl J., Unterer S. (2018). Intestinal lesions in dogs with acute hemorrhagic diarrhea syndrome associated with netF-positive *Clostridium perfringens* type A. J. Vet. Diagn. Investig..

[41] Mueller R.S., Unterer S. (2018). Adverse food reactions: Pathogenesis, clinical signs, diagnosis and alternatives to elimination diets. Vet. J..

